# Comparison of different methicillin-resistant *Staphylococcus aureus* (MRSA) typing methods when poorly distinguishable isolates are causing epidemics

**DOI:** 10.1186/1753-6561-5-S6-P176

**Published:** 2011-06-29

**Authors:** JJ Hirvonen, T Pasanen, S Mero, S-S Kaukoranta, M Vaara, J Vuopio, S Salmenlinna, P Tissari

**Affiliations:** 1 Clinical Microbiology, Vssas Central Hospital, Vaasa, Finland; 2 Clinical Microbiology, Helsinki University Central Hospital, Helsinki, Finland; 3 National Institute for Health and Welfare, Helsinki, Finland

## Introduction / objectives

Fast, accurate, and reliable strain-typing is a prerequisite in managing MRSA epidemics. In this study, we compared three different strain-typing methods.

## Methods

The usefulness of spa-typing, pulse-field gel electrophoresis (PFGE) and automated repetitive extragenic palindromic sequence-based (rep-PCR) method (DiversiLab, Bacterial Barcodes, Inc.) for typing Finnish MRSA strains was studied. A total of 110 clinical MRSA isolates collected from well-defined outbreaks in Ostrobothnia region and 96 isolates from several epidemics or sporadic sources all over Finland were analysed.

## Results

Among all studied isolates 11 different major rep-PCR groups, 20 different PFGE and 10 different spa types were formed. Geographically close strains from separate local epidemics in Ostrobothia caused difficulties in the grouping and discrepancies among the typing methods. From this materialthree minor rep-PCR groups could be formed by DiversiLab software’s overlay analysis and 4 different PFGE groups. All of these isolates were, however, undistinguishable by spa-typing.

## Conclusion

PFGE method had the highest discriminatory power, but it is a non-automated and labour intensive method. Spa-typing was unable to differentiate close strains that belong to separate epidemics. Automated rep-PCR typing represents an attractive option for accurate and rapid molecular typing of MRSA. In addition, a rep-PCR strain library can be created that aids laboratories to classify isolates and hospitals to monitor the occurrence of different types.

## Disclosure of interest

None declared.

